# The role of aetiology in determining anticoagulation effectiveness for the treatment of left ventricular thrombus

**DOI:** 10.1093/ehjcvp/pvaf091

**Published:** 2025-12-31

**Authors:** Johanna Jones, Holly Morgan, Krishnaraj Rathod, Robert O’Dowling, Christopher Pieri, Bianca Coldea, Benjamin Waters, Paul Wright, Sotiris Antoniou, Andrew Wragg, Amedeo Chiribiri, Anthony Mathur, Divaka Perera, Daniel A Jones

**Affiliations:** Centre for Cardiovascular Medicine and Devices, William Harvery Research Institute, Queen Mary University of London, London EC1M 6BQ, UK; Barts Interventional Group, Barts Heart Centre, Barts Health NHS Trust, West Smithfield, London EC1A 7BE, UK; Department of Cardiology, Guy’s and St Thomas NHS Trust Hospitals, London, UK; Centre for Cardiovascular Medicine and Devices, William Harvery Research Institute, Queen Mary University of London, London EC1M 6BQ, UK; Barts Interventional Group, Barts Heart Centre, Barts Health NHS Trust, West Smithfield, London EC1A 7BE, UK; Department of Cardiology, Guy’s and St Thomas NHS Trust Hospitals, London, UK; Department of Cardiology, Guy’s and St Thomas NHS Trust Hospitals, London, UK; Barts Interventional Group, Barts Heart Centre, Barts Health NHS Trust, West Smithfield, London EC1A 7BE, UK; Department of Pharmacy, Barts Heart Centre, Barts Health NHS Trust, London, UK; Department of Pharmacy, Barts Heart Centre, Barts Health NHS Trust, London, UK; Department of Pharmacy, Barts Heart Centre, Barts Health NHS Trust, London, UK; Barts Interventional Group, Barts Heart Centre, Barts Health NHS Trust, West Smithfield, London EC1A 7BE, UK; Department of Cardiology, Guy’s and St Thomas NHS Trust Hospitals, London, UK; Centre for Cardiovascular Medicine and Devices, William Harvery Research Institute, Queen Mary University of London, London EC1M 6BQ, UK; Barts Interventional Group, Barts Heart Centre, Barts Health NHS Trust, West Smithfield, London EC1A 7BE, UK; Department of Cardiology, Guy’s and St Thomas NHS Trust Hospitals, London, UK; Centre for Cardiovascular Medicine and Devices, William Harvery Research Institute, Queen Mary University of London, London EC1M 6BQ, UK; Barts Interventional Group, Barts Heart Centre, Barts Health NHS Trust, West Smithfield, London EC1A 7BE, UK

**Keywords:** Left ventricular thrombus, Anticoagulation, Direct oral anticoagulants, Stroke and systemic embolization, Non-ischaemic cardiomyopathy, Acute myocardial infarction

## Abstract

**Aims:**

Left ventricular (LV) thrombus is a severe complication of acute myocardial infarction (AMI) and chronic heart failure. While current guidelines support the use of direct oral anticoagulants (DOACs) as alternatives to vitamin K antagonists (VKA), their benefit across different aetiologies remains uncertain. This study aimed to compare the efficacy and safety of DOAC vs. VKA across different aetiologies of LV dysfunction.

**Methods and results:**

We conducted a multi-centre observational study including 901 patients with confirmed LV thrombus treated with either a VKA or DOAC. The primary outcome was thrombus resolution, secondary outcomes included stroke and systemic embolization (SSE), major bleeding and mortality with analyses performed by aetiology. The principal aetiologies were AMI (38.3%), ischaemic cardiomyopathy (ICM) (38.0%) and non-ischaemic cardiomyopathy (NICM) (23.7%). Overall, thrombus resolution was significantly higher in DOAC treated patients, but this was driven by the AMI sub-group (*P* = 0.018). Direct oral anticoagulant use independently predicted thrombus resolution (OR 2.0, 95% Cl 1.29–3.24, *P* = 0.010). Major bleeding events (BARC ≥3) were more common with VKA use (*P* = 0.008). Non-ischaemic cardiomyopathy had the highest SSE rate (15.3%, *P* = 0.002), which were significantly raised in those treated with DOAC (*P* < 0.001).

**Conclusion:**

The underlying aetiology of LV dysfunction significantly influences both treatment response and outcomes in patients with LV thrombus. Direct oral anticoagulant were associated with superior efficacy and safety in AMI-related LV thrombus, but were linked to increased rates of SSE in NICM. These findings highlight the importance of aetiology on LV thrombus management and the potential need for tailored approaches.

## Graphical Abstract

**Graphical Abstract:**
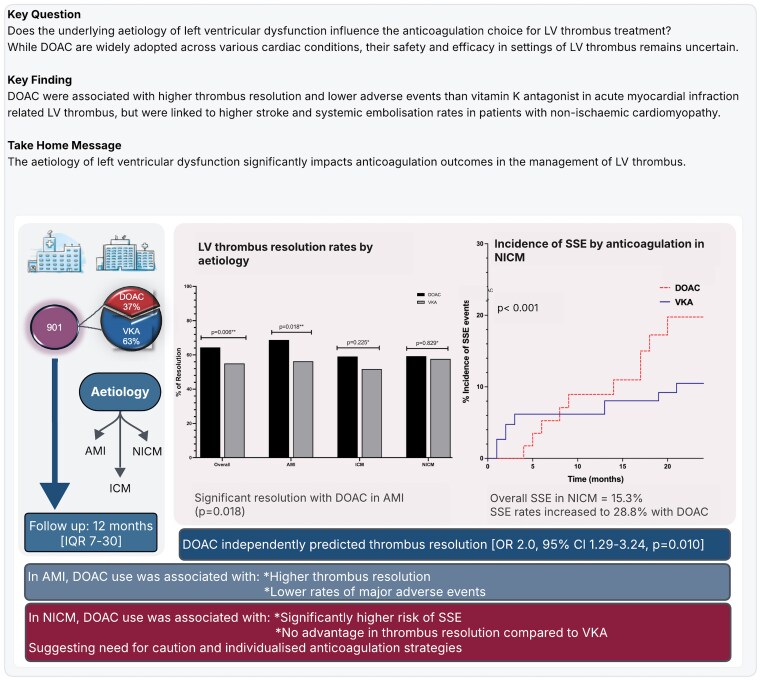

## Introduction

Left ventricular (LV) thrombus is a serious complication associated with LV dysfunction, frequently seen after acute myocardial infarction (AMI) but also in cases of chronic ischaemic (ICM) and non-ischaemic cardiomyopathies (NICM). Despite substantial advancements in the management of LV dysfunction over the past decade, the incidence of LV thrombus remains a notable clinical concern. Rates range from 4–15% in patients with AMI, with anterior MI showing a higher prevalence of up to 25%.^1-3^ While in cases of cardiomyopathies, the reported incidence ranges from 20% to 30%.^4,5^ The clinical importance of LV thrombus lies in its embolic potential, contributing to stroke, systemic embolization (SSE) and increased mortality.^2^ Both American and European guidelines recommend prompt diagnosis and anticoagulation treatment to mitigate these risks (Class IIa, level of evidence C).^6,7^ A growing body of evidence from observational studies and smaller randomized controlled trials have explored the efficacy of direct oral anticoagulants (DOAC) vs. vitamin K antagonists (VKA) for the treatment of LV thrombus, yielding promising outcomes.^8-12^ Reflecting these findings, recent guidelines now recognize DOAC as a treatment option for LV thrombus.^13,14^ However, they do not account for differences in the underlying aetiology of LV dysfunction and suggest a uniform approach to anticoagulation. Notably, evidence supporting DOAC appears strongest in AMI-related LV thrombus, with studies reporting non-inferiority to VKA in thrombus resolution.^8,9,15,16^ Conversely, data on LV thrombus in cases of ICM and NICM remains limited and inconsistent.^17-19^ Despite the accumulating evidence in support of DOAC, especially in AMI, many clinicians remain hesitant to adopt DOAC as the standard therapy, often reverting to VKA when faced with doubt regarding treatment response and safety in certain clinical contexts.^20^ This reluctance may stem from the limited number of large randomized trials and variability in outcomes across different patient populations.

Given these uncertainties, the aim of our study was to assess the impact of underlying aetiology on the safety and effectiveness of DOAC compared with VKA in the management of LV thrombus.

## Methods

### Study design and patient population

We conducted a retrospective observational study of patients with a confirmed diagnosis of LV thrombus, who presented to Barts Heart Centre and Guy’s and St Thomas’ Hospital in London, UK from 2011 to 2021. Patients were identified by screening transthoracic echocardiogram (TTE) and cardiac magnetic resonance imaging (CMR) reports. All patients with a diagnosis of LV thrombus, of any aetiology, were included in the study. Patients with no further information available were excluded.

### Left ventricular thrombus management

Patients with confirmed LV thrombus were anticoagulated with either a VKA (warfarin) or DOAC (apixaban, edoxaban, or Rivaroxaban). Direct oral anticoagulants dosing was guided by atrial fibrillation guideline recommendations and adjusted for patient-specific factors (i.e. age, renal failure). Anticoagulation selection and dosing were at the treating clinician’s discretion and taken from prescription records where available. In the DOAC group, 9 patients received a reduced dose of apixaban (2.5 mg twice daily) and 11 received a reduced dose of edoxaban (30 mg once daily) in accordance with the dose reduction criteria. The remaining patients were on standard dosing (apixaban 5 mg twice daily, rivaroxaban 20 mg once daily, and edoxaban 60 mg once daily). Data on INR and time in the therapeutic range for warfarin were inconsistently collected and therefore not analysed. In patients with AMI, triple therapy was defined as the combination of dual antiplatelet therapy (DAPT) plus an oral anticoagulant.

### Data collection

Data from both centres were collected using a research database built on common data available variables, ensuring uniform definitions, imaging parameters and outcome measures. Baseline characteristics including age, sex, ethnicity, renal disease, previous venous thromboembolic event, and cardiovascular risk factors (diabetes, hypertension, smoking history, hypercholesterolaemia, and atrial fibrillation) were collected from health records. Baseline LV ejection fraction, initial imaging modality, aetiology of LV dysfunction, choice and duration of anticoagulation, and concomitant antiplatelet therapy were recorded.

### Endpoints

Follow up data was obtained from electronic health records. Documented endpoints included bleeding events (defined by BARC criteria^21^; major bleeding classified as BARC ≥3), thromboembolic events, and death, as well as date of occurrence of these events. Stroke was defined as a neurological deficit lasting over 24 h supported by neuro-imaging; Systemic embolic events required a clinical history consistent with an acute occlusion of a peripheral artery/arteries supported by evidence of embolism from angiography, vascular imaging, or other objective testing. Events were included if a diagnosis had been confirmed by the treating team and recorded on either discharge summary (relevant ICD code) or clinic letters.

### Ethics

The study was discussed with the local ethics committees and considered exempt from formal ethical approval. The study was registered as a service evaluation at both centres (GSTT 13508, BHC 11834).

### Statistical analysis

Categorical data are summarized using absolute values (percentage). Normally distributed, continuous data are presented as mean ± standard deviation or, where skewed, as median and interquartile range (IQR) [25th to 75th centile]. Normally distributed continuous variables were compared using the Student’s *t*-tests, and the Mann–Whitney *U* test was used to compare non-normally distributed continuous variables. Categorical data were compared using the Pearson *χ*^2^ test. Long-term survival was described by the Kaplan–Meier method, and comparisons in LV thrombus resolution and survival between groups were made using the log-rank and Cox regression statistics. A two-sided *P*-value <0.05 was considered statistically significant. Multivariable cox models were adjusted for relevant co-factors including age, sex, EF, renal function, thrombus size, underlying aetiology, atrial fibrillation, and DAPT. All statistical analyses were performed using SPSS version 29.0 (SPSS Inc.) or GraphPad Prism version 9.0.

## Results

### Baseline demographics

**Figure 1:**
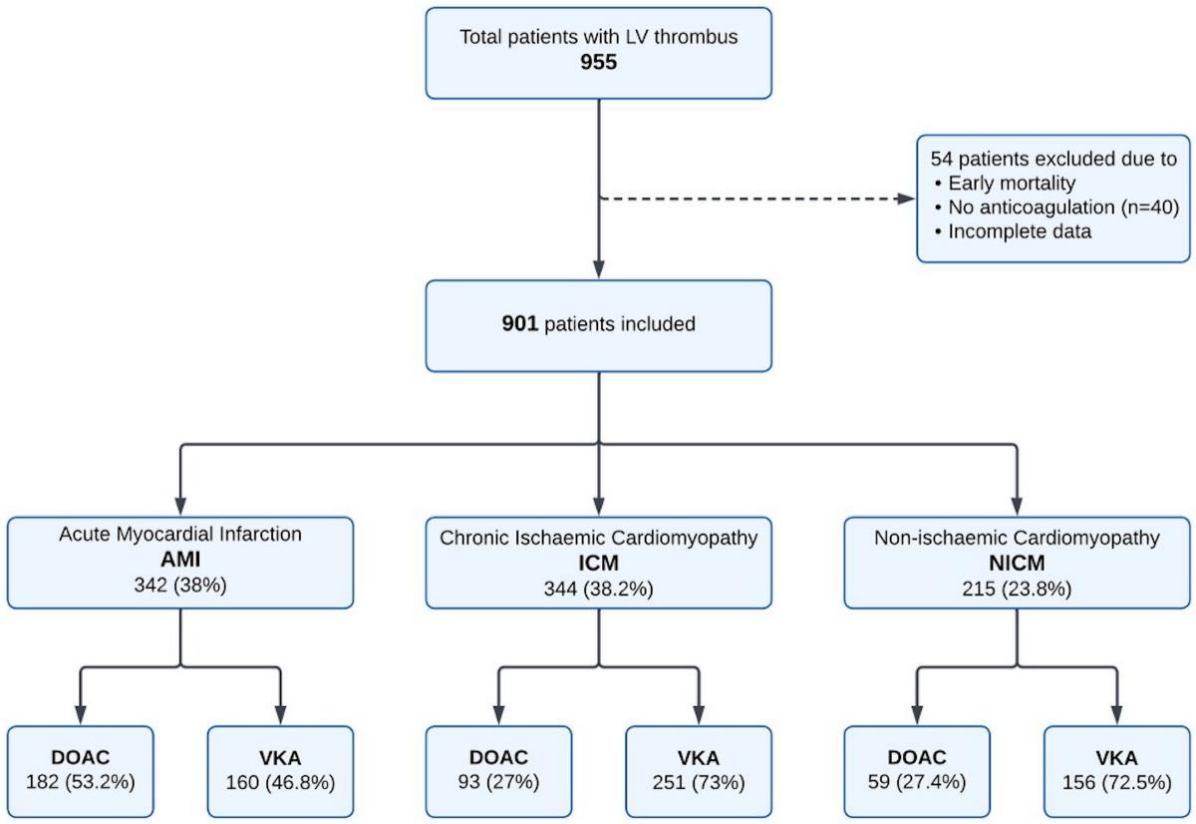
Flow diagram of patients with left ventricular thrombus based on aetiology and treatment.

The study identified 955 patients diagnosed with LV thrombus, of which 901 received treatment with either a VKA (567 patients, 63%) or a DOAC (334 patients, 37%). Forty patients were not anti-coagulated with either drug due to early mortality or clinician decision against anticoagulation, whereas fourteen were excluded due to incomplete data. rivaroxaban (42.6%) was the most frequently prescribed DOAC followed by apixaban (36.7%) and edoxaban (20.7%). LV thrombus was primarily identified by CMR (53.5%) over TTE (46.5%). Primary aetiologies for LV thrombus were AMI (38.0%), ICM (38.2%), and NICM (23.8%) (*Figure 1*). Among NICM patients, 80.9% had dilated cardiomyopathy, with the remainder attributed to hypertrophic, eosinophilic or peripartum cardiomyopathies.

Baseline characteristics were largely similar between the VKA and DOAC groups, aside from a higher prevalence of hypertension in the DOAC group (59.7% vs. 49.9%, *P* = 0.005) and a lower initial ejection fraction in the VKA group (33.6 ± 13.0 vs. 35.2 ± 11.2 *P* < 0.001) (*Table 1*). Of the entire cohort, 271 patients (30.2%) were discharged on triple therapy for a median of 3 months (1–3 months). The remaining patients were managed with either a single anti-platelet agent plus anticoagulation (34.2%) or anticoagulation monotherapy (35.6%). Triple therapy was more prevalent in the DOAC group (47.1% vs. 20.2%), whereas VKA was favoured with single anti-platelet therapy (40.6% vs. 23.4%) or as monotherapy (39.2% vs. 29.4%; *P* < 0.001).

**Table 1:** Baseline characteristics of patients with left ventricular thrombus comparing VKA vs. DOAC

	VKA (*N* = 567)	DOAC (*N* = 334)	*P* value
Age (mean ± SD)	60.79 ± 13.7	59.93 ± 13.4	0.755
Sex (male)	466 (82.2%)	271 (81.1%)	0.693
Medical history			
Current/ex-smoker	264 (50.9%)	176 (56.1%)	0.146
Hypercholesterolaemia	227 (42.4%)	123 (38.9%)	0.450
Hypertension	269 (49.9%)	190 (59.7%)	0.005**
Diabetes mellitus	124 (23.1%)	78 (24.7%)	0.597
Previous PCI	187 (34.4%)	94 (29.2%)	0.111
Atrial fibrillation	68 (12.6%)	45 (14.2%)	0.510
History of VTE	56 (10.5%	24 (7.6%)	0.170
Renal failure	82 (15.3%)	45 (14.2%)	0.657
LVEF (mean ± SD)	33.59 ± 13	35.15 ± 11.2	<0.001***
Thrombus size (≥2 cm)	203 (37.5%)	100 (30.9%)	0.049*
Anticoagulation			
Warfarin	554 (100%)	0	
Rivaroxaban	0	148 (42.6%)	
Apixaban	0	127 (36.7%)	
Edoxaban	0	72 (20.7%)	
Additional anti-platelets			
Dual anti-platelets	114 (20.2%)	157 (47.1%)	<0.001***
Single anti-platelets	229 (40.6%)	78 (23.4%)	
Anti-coagulation only	221 (39.2%)	98 (29.4%)	

^*^ *P <* 0.05; **P < 0.01; ***P < 0.001.

DOAC, direct oral anticoagulation; PCI, percutaneous coronary intervention; VKA, vitamin K antagonist; VTE, venous thromboembolism.

### Characteristics by aetiology

Direct oral anticoagulants were predominantly used in AMI cases (53.2%), whereas VKA were preferred in ICM (73.0%) and NICM (72.5%), which corresponded to the higher rates of DAPT use in the DOAC group. Other findings included NCIM patients being younger (56.0 ± 15.8 years) compared with AMI (60.0 ± 12.5) and ICM (63.8 ± 12.2), *P* < 0.001. Atrial fibrillation was more frequently observed in NICM (19.5%) and ICM (16.9%) compared with AMI (6.0%), *P* < 0.001. While AMI patients had a relatively better ejection fraction (36.7 ± 10.5) compared with ICM (34.6 ± 11.0) and NICM (29.5 ± 15.6), *P* < 0.001, they were more likely to exhibit larger thrombi (*P* = 0.002) (see Supplementary material online, *Table S1*).

### Anticoagulation choice based on aetiology

In the AMI group, patients with a lower LV ejection fraction were more likely to receive VKA (35.7 ± 11.7 vs. 37.5 ± 9.4, *P* = 0.004). Vitamin K antagonists was also preferred compared with DOAC in cases of larger thrombi in both ICM (34.6% vs. 22.0%, *P* = 0.027) and NICM (34.9% vs. 4.3%, *P* = 0.007) (*Table 2*).

**Table 2:** Baseline characteristics of patients with left ventricular thrombus stratified by anticoagulation within each aetiology

	AMI			ICM			NICM		
	VKA *n* = 160	DOAC *n* = 182	*P* value	VKA *n* = 251	DOAC *n* = 93	*P* value	VKA *n* = 156	DOAC *n* = 59	*P* value
Age	60.88 ± 13	59.25 ± 12.1	0.549	64.27 ± 12	62.47 ± 12.8	0.435	55.12 ± 15.2	58.39 ± 17.2	0.228
Sex	135 (84.4%)	142 (78.0%)	0.135	216 (86.1%)	84 (90.3%)	0.293	115 (73.7%)	45 (76.3%)	0.702
Medical history									
Current/ex-smoker	96 (60.0%)	116 (63.8%)	0.817	114 (45.4%)	44 (47.3%)	0.689	54 (34.6%)	16 (27.1%)	0.351
Hypercholesterolaemia	70 (43.8%)	80 (43.9%)	0.937	135 (53.7%)	39 (41.9%)	0.196	22 (14.1%)	4 (6.7%)	0.162
Hypertension	72 (45.0%)	108 (59.3%)	0.013*	143 (56.9%)	59 (63.4%)	0.117	54 (34.6%)	23 (38.9%)	0.432
Diabetes mellitus	33 (20.6%)	50 (27.9%)	0.161	65 (25.9%)	25 (26.9%)	0.695	26 (16.7%)	3 (5.1%)	0.031*
Atrial fibrillation	11 (7.0%)	9 (5.0%)	0.444	37 (14.7%)	17 (18.3%)	0.308	20 (12.8%)	19 (32.2%)	<0.001***
History of VTE	17 (10.6%)	12 (6.7%)	0.168	20 (8.0%)	6 (6.5%)	0.708	19 (12.2%)	6 (10.7%)	0.747
Renal failure	21 (13.1%)	27 (14.9%)	0.686	34 (13.5%)	12 (12.9%)	0.980	27 (17.3%)	6 (10.7%)	0.193
LVEF (mean ± SD)	35.72 ± 11.7	37.49 ± 9.4	0.004**	35.06 ± 11.4	33.43 ± 9.8	0.118	29.05 ± 15.6	30.66 ± 15.8	0.565
Thrombus size (≥2 cm)	69 (43.1%)	71 (39.0%)	0.474	81 (32.3%)	20 (22.0%)	0.027*	53 (33.9%)	9 (15.2%)	0.007**

^*^ *P* < 0.05; **P < 0.01; ***P < 0.001.

AMI, acute myocardial infarction; DOAC, direct oral anticoagulation; ICM, chronic ischaemic cardiomyopathy; NICM, non-ischaemic cardiomyopathy; PCI, percutaneous coronary intervention; VKA, vitamin K antagonist; VTE, venous thromboembolism.

### Thrombus resolution

**Figure 2:**
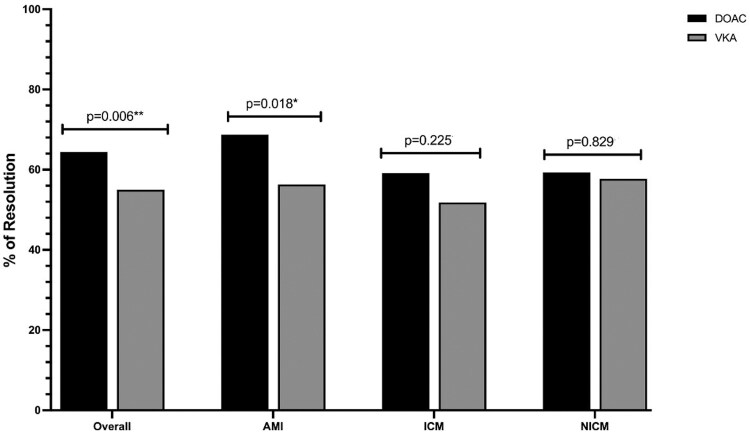
Left ventricular thrombus resolution rates by aetiology—bar graph comparing the rates of left ventricular thrombus resolution between direct oral anticoagulants vs. vitamin K antagonists across aetiologies.

Imaging was available for 867 patients (96.2%) over a median follow-up period of 12 months (IQR 7–30). Cardiac magnetic resonance was utilized in 61.7% of cases, predominantly in chronic ischaemic (67.2%) and non-ischaemic cardiomyopathy (70.7%), while TTE was frequently used in AMI cases (69.6%), *P* < 0.001. Overall, rates of thrombus resolution were significantly higher in the DOAC group compared with those treated with VKA, *P* = 0.006. Subgroup analysis revealed that DOAC achieved superior thrombus resolution in AMI patients (*P* = 0.018), but showed no difference in ICM (*P* = 0.225) or NICM (*P* = 0.829) (*Figure 2*).

### Predictors of thrombus resolution

Logistic regression was performed to identify independent predictors of LV thrombus resolution on first follow up imaging. Variables included in the model were age, sex, aetiology of LV impairment, renal disease, LV ejection fraction, DAPT use, thrombus size, and DOAC use. Direct oral anticoagulants use was a positive predictor of thrombus resolution (OR 2.0, 95% CI 1.29–3.24, *P* = 0.010), whilst a larger thrombus size at diagnosis was associated with LV thrombus persistence (OR: 0.49: 95% CI 0.32–0.76, *P* = 0.001). The aetiology for LV dysfunction was not found to be an independent predictor of thrombus resolution from the model (*P* = 0.090).

### Major clinical outcomes

**Figure 3:**
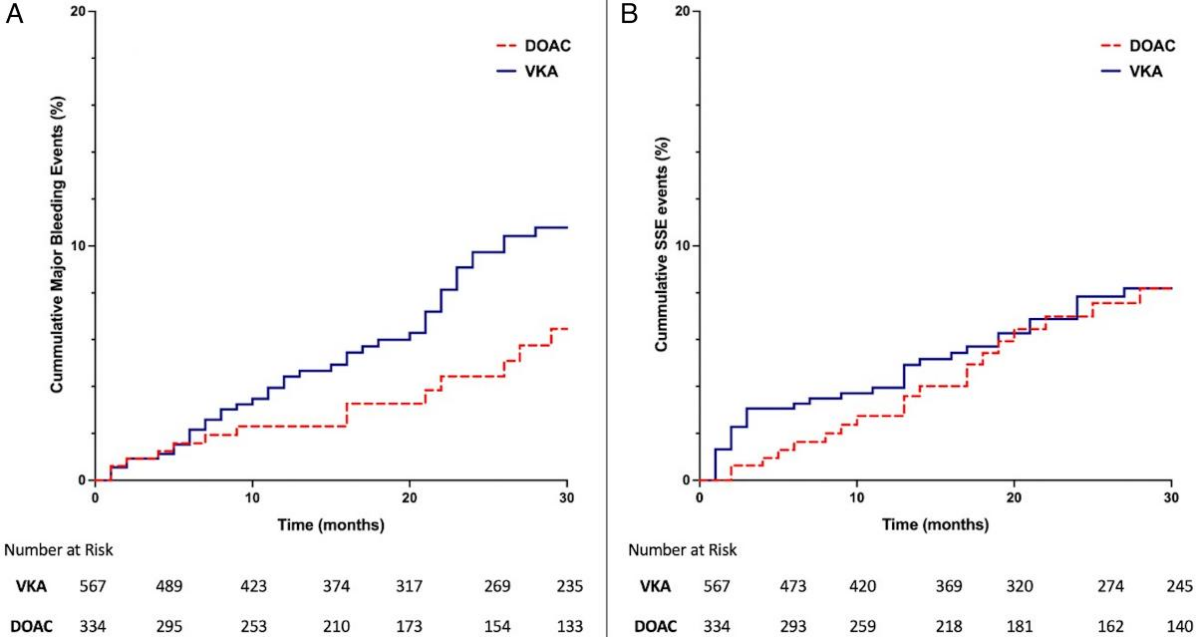
K-M curves showing the cumulative incidence of major bleeding and stroke and systemic embolization events by type of anticoagulation.

**Figure 4:**
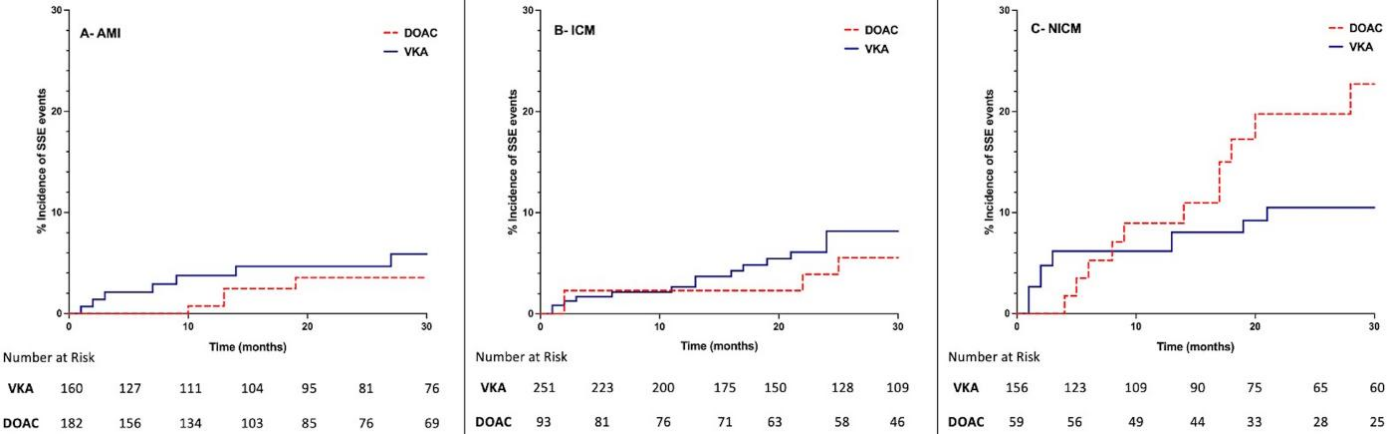
Incidence of stroke and systemic embolization events stratified by aetiology and anticoagulation treatment.

Overall, SSE and major bleeding occurred in 10.0% and 6.4% of patients, respectively. Patients treated with VKA experienced higher major bleeding rates (8.1%) compared with those on DOAC (3.6%), *P* = 0.008. Stroke, systemic embolization rates were comparable across both groups (DOAC- 9.6% and VKA- 10.4%), *P* = 0.691 (*Figure 3*). But were highest in NICM, with rates of 15.3% compared with 10.8% in ICM and 6.1% in AMI, *P* = 0.002. Within NICM, DOAC use was significantly associated with higher SSE rates compared with VKA (28.8% vs. 10.3%, *P* < 0.001). This difference in SSE events by anticoagulation type was not observed in patients with AMI (*P* = 0.152) or ICM (*P* = 0.239) (*Figure 4*). No difference in major bleeding was noted amongst the aetiologies, *P* = 0.473. Of note, the overall mortality rate was 12.2%, with VKA treated patients experiencing higher mortality compared with DOAC (14.1% vs. 9%, *P* = 0.023). Mortality rates were highest in NICM (15.8%), followed by ICM (13.7%) and AMI (8.5%), *P* = 0.021.

### Predictors of Major outcomes

Cox regression analysis was conducted to identify predictors for each of the major outcomes.

#### Stroke, systemic embolization

Independent predictors were NICM (HR 1.63, 95% Cl 1.22–2.19, *P* < 0.001) and a larger thrombi at diagnosis (HR 1.81, 95% Cl 1.17–2.79, *P* = 0.007).

#### Major bleeding

The likelihood of major bleeding increased with advancing age (HR 1.02, 95% Cl 1.00–1.05, *P* = 0.028), and those treated with VKA (HR 0.47, 95% Cl 0.24–0.94, *P* = 0.032).

#### Mortality

Independently associated with advancing age (HR 1.06, 95% Cl 1.04–1.07, *P* < 0.001), severe LV dysfunction (HR 0.96, 95% Cl 0.95–0.98, *P* < 0.001), and NICM (HR 1.32, 95% Cl 1.03–1.69, *P* = 0.026).

### Non-anticoagulated patient outcomes

Although not included in the primary comparative analysis, the outcomes of patients who did not receive oral anticoagulation remain clinically relevant. A total of 40 patients with LV thrombus did not receive oral anticoagulation, 32 received subcutaneous heparin, and 8 were on antiplatelet therapy only. This group was generally older and had higher rates of comorbidities. Follow up imaging, available for 45% of these patients, indicated a 20.8% thrombus resolution rate, predominantly in the AMI cohort. Notably, this group exhibited high rates of SSE (12.5%), major bleeding (10%), and mortality (63%).

## Discussion

This large observational study highlights the intricate relationship between the aetiology of LV dysfunction, anticoagulation choice and the resulting clinical outcomes in patients diagnosed with LV thrombus. Our findings emphasize the importance of tailoring anticoagulation therapy to the underlying cause of LV dysfunction, as both efficacy and safety vary considerably depending on the clinical presentation. We show DOAC as a safe and an independent predictor of LV thrombus resolution, reinforcing previous studies that suggest they are at least as effective as VKA while offering a better safety profile.^10-12,22,23^ A key observation from our study was the superior performance of DOAC over VKA in achieving thrombus resolution in AMI-related LV thrombus. However, this benefit did not extend to patients diagnosed with LV thrombus in cases of chronic ICM and NICM. Of particular concern, DOAC use in the NICM cohort was associated with the highest stroke and systemic embolization rates, emphasizing the need for caution in this group.

In the AMI cohort, DOAC were predominantly prescribed and were particularly effective in achieving thrombus resolution alongside a lower incidence of thromboembolic events. Patients in this group achieved earlier thrombus resolution despite presenting with a larger thrombi at diagnosis. This may reflect the recovery of LV function following percutaneous coronary intervention and resolution of myocardial stunning. Although DAPT was frequently used in the AMI cohort and may have contributed in thrombus resolution, our multivariate analysis did not identify antiplatelet use or LV ejection fraction as independent predictors. Importantly, DOAC use was associated with lower SSE and major bleeding compared with VKA. These findings align with prior studies that focused exclusively on AMI-related LV thrombus formation.^8,9,12,16,24^ Recent meta-analysis also support this observation, reporting nearly twofold higher thrombus resolution and up to 70% lower thromboembolic events with DOAC compared with VKA in AMI patients.^25^ Collectively, evidence supports the notion that DOAC are a favourable option in AMI, combing safety with efficacy.

In contrast, our findings raise concern regarding DOAC use in patients with LV thrombus secondary to NICM. This group exhibited the highest rates of SSE (15.3%), which increased to 28.8% in those treated with DOAC compared with VKA. Our results are comparable to Robinson et al, who observed elevated SSE rates in patients treated with DOAC, notably the majority of their cohort were composed of chronic ischaemic and dilated cardiomyopathy.^17^ Likewise, Fujino *et al*.^26^ also reported higher rates of thromboembolism in DCM patients with LV thrombus compared with other cardiomyopathies, including ICM. In our NICM cohort, thrombus resolution rates did not differ significantly between DOAC and VKA. One possible explanation is that the mechanisms underlying thrombus formation in this cohort, such as chronic endothelial dysfunction and a sustained prothrombotic state inherent to long-standing heart failure, may attenuate the efficacy of DOAC. A history of atrial fibrillation was frequent in this group, raising the possibility that LV thrombus development occurred whilst already established on an anticoagulation. Although DOACs are standard therapy for stroke prevention in non-valvular atrial fibrillation and are effective for left atrial appendage thrombus, LV thrombus likely differs in its pathogenesis, which may translate to a different therapeutic response. Current AHA and ESC guidelines recognize DOAC therapy as a reasonable alternative to VKA for post-MI LV thrombus, but emphasize the limited and heterogeneous evidence in chronic ICM and NICM. Our findings, therefore, are aligned with this cautious perspective and reinforce that one anticoagulation strategy does not fit all aetiologies.

Patients with ICM demonstrated intermediate outcomes, with SSE rates of 10.8%. In this group, we observed no significant differences in thrombus resolution or adverse events between the type of anticoagulation used. Suggesting that unlike in AMI, the choice of anticoagulant may be less critical in ICM, or that the disease characteristics and thrombus formation mechanisms in this group could render both treatment options equally effective.

The overall mortality and SSE rates in our cohort following the diagnosis of LV thrombus were 12% and 10%, respectively, which are consistent with findings from prior observational studies and meta-analyses.^5,27^ We also identified several predictors of adverse outcomes in patients with LV thrombus. Thromboembolic events are likely to occur in patients with larger thrombi at diagnosis and in NICM, whilst major bleeding is more likely to occur in older patients and those on VKA therapy. Additionally, mortality was independently associated with advancing age, severe LV dysfunction and NICM. Highlighting the need to balance benefits of thrombus resolution against bleeding risk, particularly in older or high-risk patients, and to individualize anticoagulant choice based on aetiology and clinical profile.

Our study adds to the growing body of evidence that DOAC are a pragmatic and effective alternative to VKA in AMI-related LV thrombus, while non-ischaemic cases warrant caution and further investigation. Contemporary guideline statements and expert consensus recommend anticoagulation until imaging confirms resolution, typically for 3–6 months, but fall short of universally endorsing one anticoagulant over the other due to the absence of randomized data. Given the retrospective, observational nature of our registry, causality cannot be inferred. But mechanistic differences in thrombus genesis and progression likely underpin the divergent responses to anticoagulation observed across aetiologies. Consequently, these results reinforce the need for prospective, aetiology-stratified trials beyond the post-infarct setting to define the optimal anticoagulant choice for LV thrombus.

### Limitations

The limitations of this study lies in its retrospective and non-randomized nature, which introduces potential biases, such as clinician preference influencing treatment choice, dosing and timing of follow-up imaging. Although imaging was performed and reported by specialist cardiologists at both centres, heterogeneity in imaging modality and absence of central core laboratory adjudication could influence results. We attempted to account for confounders through multivariable adjustment, yet residual factors may remain unmeasured.

Formal bleeding risk scores were not calculated, as baseline data such as prior bleeding, liver disease and alcohol use was not routinely collected. Minor bleeding events were likely under ascertained. Differences in concomitant antiplatelet therapy may have affected SSE and thrombus resolution rates. Furthermore, there is a possibility of incomplete follow up due to local hospital presentations that may not have been captured by review of the tertiary hospital’s electronic record systems. Mortality is linked to national NHS hospital episode statistics data and therefore would be expected to be contemporary and accurate. Hospital presentations however, including those of SSE or bleeding events and medication compliance, are more difficult to collect remotely. Therefore the other elements of our primary outcome may be underestimated. This was avoided as much as possible by reviewing all outpatient clinic letters and follow up imaging requests to attempt to capture any documented events that may have been missed. With regards to VKA dosing, INR and time-in-therapeutic range were not consistently recorded and could not be analysed. These limitations reflect real-world variability and underscore the need for prospective studies.

## Conclusion

In a large multi-centre registry of LV thrombus, DOAC offered favourable outcomes, with lower thromboembolism and major bleeding events compared with VKA in patients with AMI. However, the elevated stroke and systemic embolization rates in NICM patients treated with DOAC highlights the likely influence of aetiology on treatment outcomes and the need for tailored anticoagulation strategies. Future studies are warranted to identify the appropriate anticoagulation type across different patient groups in order to enhance thrombus resolution whilst minimizing adverse events.

## Supplementary Material

pvaf091_Supplementary_Data

## Data Availability

Data that support the findings of this observational study are available upon request from the corresponding author.
