# Supplementary material for: The role of aetiology in determining anticoagulation effectiveness for the treatment of left ventricular thrombus
Source: Eur Heart J Cardiovasc Pharmacother. 2025 Dec 31;12(2):88–96. doi: 10.1093/ehjcvp/pvaf091 (PMC12946968; doi:10.1093/ehjcvp/pvaf091)
Supplement: pvaf091_Supplementary_Data [file pvaf091_supplementary_data.docx]

**Supplementary Tables**

|  | AMI  (n=342) | ICM  (n=344) | NICM  (n=215) | p value |
| --- | --- | --- | --- | --- |
| Age (mean+/-SD) | 60 ± 12.5 | 63.78 ± 12.2 | 56.02 ± 15.8 | <0.001 |
| Sex (Male) | 277 (81%) | 300 (87.2%) | 160 (74.4%) | <0.001 |
|  | | | | |
| Medical History | | | | |
| Current/Ex-Smoker | 212 (64.2%) | 158 (51.1%) | 70 (36.1%) | <0.001 |
| Hypercholesterolaemia | 150 (44.6%) | 174 (54.2%) | 26 (13.3%) | <0.001 |
| Hypertension | 180 (53.7%) | 202 (62.3%) | 77 (38.9%) | <0.001 |
| Diabetes mellitus | 83 (24.9%) | 90 (27.8%) | 29 (14.9%) | 0.003 |
| Atrial Fibrillation | 20 (6%) | 54 (16.9%) | 39 (19.5%) | <0.001 |
| History of VTE | 29 (8.7%) | 26 (8.1%) | 25 (12.8%) | 0.173 |
| Renal Failure | 48 (14.2%) | 46 (14.4%) | 33 (16.8%) | 0.693 |
|  | | | | |
| LVEF (mean +/-SD) | 36.67 ± 10.56 | 34.62 ± 11.04 | 29.50 ± 15.6 | <0.001 |
| Thrombus size (≥2cm) | 140 (42.2%) | 101 (31.1%) | 62 (29.7%) | 0.002 |
|  | | | | |
| Anticoagulation | | | | <0.001 |
| VKA (Warfarin) | 160 (46.8%) | 251 (73%) | 156 (72.5%) |  |
| DOAC | 182 (53.2%) | 93 (27%) | 59 (27.4%) |  |
|  |  |  |  |  |
| Additional Antiplatelets | | | | <0.001 |
| Dual | 271 (79.7%) | 0 | 0 |  |
| Single | 55 (16.2%) | 211 (61.5%) | 41 (19.2%) |  |
| Anticoagulation only | 14 (4.1%) | 132 (38.5%) | 173 (80.8%)  AMI- Acute myocardial infarction, ICM- Chronic ischaemic cardiomyopathy, NICM- Non-ischaemic cardiomyopathy, VKA- Vitamin K antagonist, DOAC- Direct oral anticoagulation, PCI- Percutaneous coronary intervention, VTE- Venous thromboembolism. |  |

**Table S1: Baseline characteristics of patients with Left ventricular thrombus stratified by aetiology**
